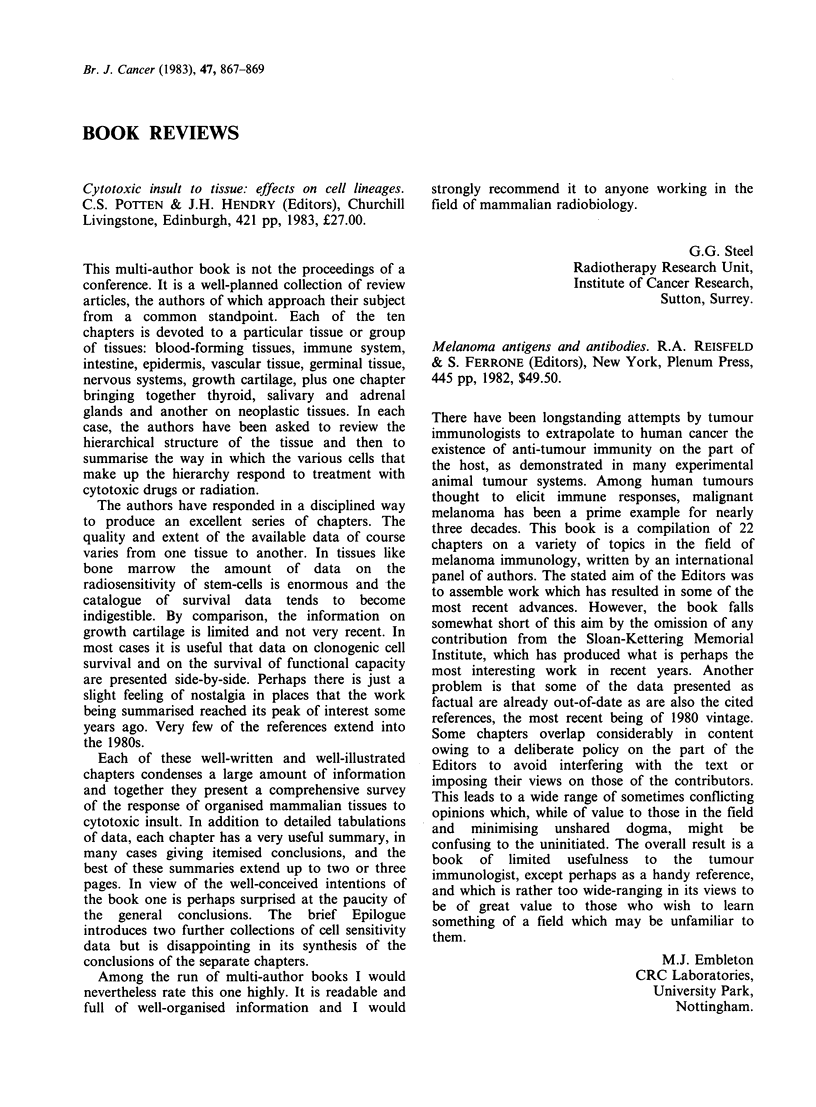# Melanoma antigens and antibodies

**Published:** 1983-06

**Authors:** M.J. Embleton


*Melanoma antigens and antibodies.* R.A. REISFELD & S. FERRONE (Editors), New York, Plenum Press, 445 pp, 1982, $49.50.

There have been longstanding attempts by tumour immunologists to extrapolate to human cancer the existence of anti-tumour immunity on the part of the host, as demonstrated in many experimental animal tumour systems. Among human tumours thought to elicit immune responses, malignant melanoma has been a prime example for nearly three decades. This book is a compilation of 22 chapters on a variety of topics in the field of melanoma immunology, written by an international panel of authors. The stated aim of the Editors was to assemble work which has resulted in some of the most recent advances. However, the book falls somewhat short of this aim by the omission of any contribution from the Sloan-Kettering Memorial Institute, which has produced what is perhaps the most interesting work in recent years. Another problem is that some of the data presented as factual are already out-of-date as are also the cited references, the most recent being of 1980 vintage. Some chapters overlap considerably in content owing to a deliberate policy on the part of the Editors to avoid interfering with the text or imposing their views on those of the contributors. This leads to a wide range of sometimes conflicting opinions which, while of value to those in the field and minimising unshared dogma, might be confusing to the uninitiated. The overall result is a book of limited usefulness to the tumour immunologist, except perhaps as a handy reference, and which is rather too wide-ranging in its views to be of great value to those who wish to learn something of a field which may be unfamiliar to them.

M.J. Embleton
CRC Laboratories,
University Park,
Nottingham.